# 501. Risk Factors for Persistent Active COVID-19 Among Patients Receiving B-Cell Depleting Therapy versus Non-B-Cell Immunosuppression

**DOI:** 10.1093/ofid/ofad500.570

**Published:** 2023-11-27

**Authors:** Kevin Voll, Jacob Kartes, Emily S Spivak, Hannah Imlay

**Affiliations:** University of Utah School of Medicine - - Salt Lake City, UT, Los Angeles, California; Intermountain Health, Salt Lake City, Utah; University of Utah School of Medicine, Salt Lake City, UT; University of Utah Health, Salt Lake City, UT

## Abstract

**Background:**

Cases of COVID-19 characterized by prolonged replication of SARS-CoV-2 (persistent active COVID [PAC]) have been reported among immunocompromised patients, particularly those receiving B-cell depleting therapy (BCDT); however, the risk has not been quantified. We aimed to identify risk factors associated with PAC, including quantifying the risk associated with receipt of BCDT vs. other types of immunosuppression.

**Methods:**

We retrospectively identified four groups of immunocompromised patients who tested positive for SARS-CoV-2 between 4/01/2020 and 4/30/2021. These groups included one group of patients who received an anti-CD20 BCDT within 1 year before a positive SARS-CoV-2 PCR and three groups who received non-BCDT which included hematopoietic cell transplant (HCT) recipients, solid organ transplant (SOT) recipients, and patients receiving chemotherapy for hematologic malignancies (HM). Chart review was performed to characterize the clinical course and development of PAC, which was defined as any of the following: (1) initial clinical improvement followed by progression of illness extending beyond 14 days, characterized by persistent fevers or progressive respiratory failure; or (2) persistent symptoms with demonstration of absent seroconversion ≥ 14 days into illness. This distinction may be supported by low crossing thresholds that occurred ≥ 14 days into illness. Logistic models were performed to identify factors associated with development of PAC and other COVID outcomes.

**Results:**

We identified 67 COVID-positive patients who had received BCDT as well as 30 HM, 118 SOT, and 29 HCT patients. PAC occurred in 15/67 (22%) of patients receiving BCDT vs. 3/177 (1.7%) patients (all SOT recipients) in the non-BCTD group (SOT, HCT, HM) (Odds Ratio, 16.7; 95% confidence interval [CI] 4.7 to 60.0; p=0.00005). 30-day hospitalization and 90-day mortality were similar in BCDT and non-BCDT groups (Table 1, Figure 1) (p > 0.05).

Table 1.

**Figure:**
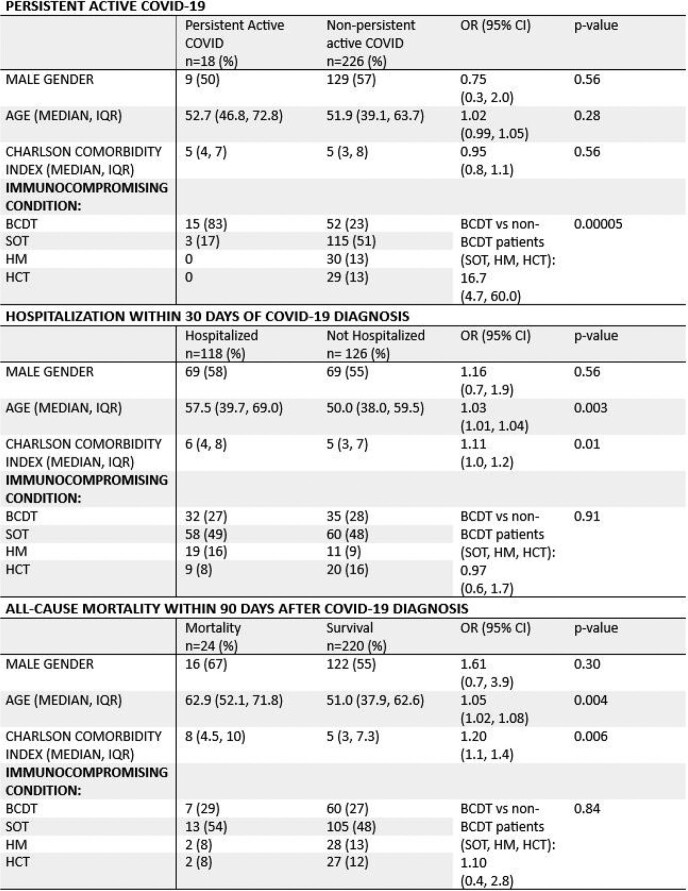

Risk factors for specific outcomes of COVID-19

Figure 1.

**Figure:**
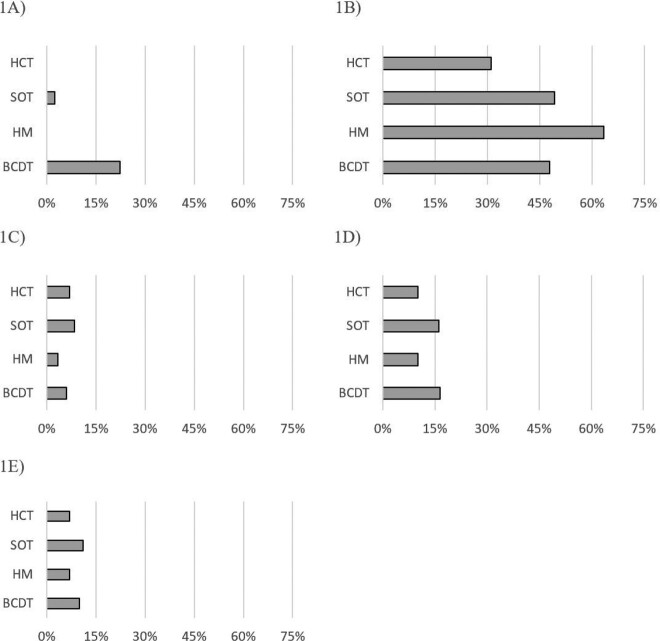

Proportion of patients from each cohort of immunocompromised patients who developed persistent active COVID (1A), required hospitalization within 30 days (1B), required mechanical ventilation (1C), required ICU admission (1D), or died within 90 days after diagnosis of COVID-19 (1E)

**Conclusion:**

Receipt of BCDTs was significantly associated with the development of PAC in comparison to other forms of severe immunosuppression. However, rates of severe outcomes associated with COVID-19 were similar across all groups. More research is necessary to determine effective prevention or treatment strategies for PAC.

**Disclosures:**

**All Authors**: No reported disclosures

